# Proximate, Antinutrients and Mineral Composition of Raw and Processed (Boiled and Roasted) *Sphenostylis stenocarpa* Seeds from Southern Kaduna, Northwest Nigeria

**DOI:** 10.1155/2014/280837

**Published:** 2014-03-16

**Authors:** Uche Samuel Ndidi, Charity Unekwuojo Ndidi, Abbas Olagunju, Aliyu Muhammad, Francis Graham Billy, Oche Okpe

**Affiliations:** Department of Biochemistry, Ahmadu Bello University, Zaria, Kaduna 810001, Nigeria

## Abstract

This research was aimed at evaluating the proximate composition, level of anti-nutrients, and the mineral composition of raw and processed *Sphenostylis stenocarpa* seeds and at examining the effect of processing on the parameters. From the proximate composition analysis, the ash content showed no significant difference (*P* > 0.05) between the processed and unprocessed (raw) samples. However, there was significant difference (*P* < 0.05) in the levels of moisture, crude lipid, nitrogen-free extract, gross energy, true protein, and crude fiber between the processed and unprocessed *S. stenocarpa*. Analyses of the antinutrient composition show that the processed *S. stenocarpa* registered significant reduction in levels of hydrogen cyanide, trypsin inhibitor, phytate, oxalate, and tannins compared to the unprocessed. Evaluation of the mineral composition showed that the level of sodium, calcium, and potassium was high in both the processed and unprocessed sample (150–400 mg/100 g). However, the level of iron, copper, zinc, and magnesium was low in both processed and unprocessed samples (2–45 mg/100 g). The correlation analysis showed that tannins and oxalate affected the levels of ash and nitrogen-free extract of processed and unprocessed seeds. These results suggest that the consumption of *S. stenocarpa* will go a long way in reducing the level of malnutrition in northern Nigeria.

## 1. Introduction

Legumes are staple foods for many people in different parts of the world. The seeds have an average of twice as much protein as cereals by percentage and usually contain more balanced profile of essential amino acids [1]. They range from the highly utilized legumes such as soybean, groundnut, and cowpea to the lesser known ones like* Sphenostylis stenocarpa*,* Mucuna cochinchinensis*, and* Mucuna flagellipes*.

African Yam Bean (AYB),* Sphenostylis stenocarpa*, is a grain legume cultivated in Central African Republic, Zaire, East Africa, and Ethiopia for its tubers and in the southeastern Nigeria for its edible seeds [2]. It is believed to be one of the most important tuberous legumes in Africa [2]. The seed grains and tubers are the two major organs of immense economic importance as food for Africans. However, there are cultural and regional preferences. In West Africa, the seeds are preferred to the tubers but tubers are relished in East and Central Africa [3]. In Southeastern Nigeria, the seeds are roasted and eaten with kernel seed. In addition, the seeds are also eaten as porridge when prepared with yam. It has been discovered that the plant is also found in some parts of Southern Kaduna, northwestern part of Nigeria where it is called “Majingba.” The Chalas, an ethnic group in the Nkwanta District, Ghana, boil the dry seeds for about three hours, replacing the water intermittently. The cooked beans are made into a sauce and eaten with “gari”, a roasted cassava product.

Several authors have evaluated the chemical composition of the AYB [2, 4, 5]. According to Evans and Boulter [6], it contains high lysine levels while both methionine and tryptophan contents are low. Duke et al. [7] showed that both the lysine and methionine contents of the protein are equal to or better than those of soybean. It also has high metabolic energy, low true protein digestibility, moderate mineral content, and amino acid content that compares favorably with most pulses [8]. Its fatty acid composition is similar to most of the common edible pulses [8]. Uguru and Madukaife [9] evaluated the nutritional genotype of 44 African Yam Beans and reported that the crop is well balanced in essential amino acids and has higher amino acid levels than pigeon pea, cowpea, and Bambara nut.

In spite of the fact that several works have been conducted on the African Yam Bean, consumers have not been showing an increasing interest in AYB seed because of limited information on their nutritional qualities and potential health benefit. As a matter of fact, AYB is in danger of extinction because of the high premium placed on the major legumes such as soya bean, cowpea, groundnut,and lima bean [10]. In addition, its current low status means that the potential is largely unexploited and it receives little research attention in Ghana [10].

Studies have, however, shown that the lesser known legumes together with other conventional legumes can be used in combating malnutrition prevalent in the third world. Therefore, research on such crops merits significant consideration. This research was undertaken to investigate the effects of processing on the nutrient and antinutrient constituents and the mineral composition of AYB seed.

## 2. Materials and Methods

### 2.1. Materials

The African Yam Beans seeds were obtained from farmers in Fadan Ninzo, Randa, and Gwantu villages, in Sanga Local Government Area, Kaduna South, Kaduna State, northwest Nigeria in September, 2011. The seed was identified and authenticated at the Herbarium unit of the Department of Agronomy, Ahmadu Bello University, Zaria, Nigeria, where a voucher specimen (1109) was deposited. The fresh seeds were divided into two categories: unprocessed (raw) and processed (boiled and roasted). The standards for minerals were purchased from Merck, India.

### 2.2. Raw (Unprocessed)* Sphenostylis stenocarpa*

The fresh seeds were thoroughly cleaned, and any foreign materials and broken and immature seeds were removed and shade dried for three (3) consecutive days to constant weight at room temperature (25–30°C). The seeds were pulverized to fine particle using the laboratory mill (SPI Supplies, PA, USA) and then stored in an airtight container at 4–6°C until they were used.

### 2.3. Processing Techniques

#### 2.3.1. Boiling

A set of AYB seeds (100 g) were boiled in distilled water (100°C) in a bean: water ratio of 1 : 10 (w/v) for 3 h and 48 min, which is the established time frame known for it to be ready as practiced by the locals. After boiling, the water was drained off and the boiled sample was mashed into paste using a ceramic mortar and then stored in an airtight container at 4–6°C until it was used.

#### 2.3.2. Roasting

Another set of AYB seeds (100 g) placed on frying pan were roasted using firewood for 1 h at about 300°C as practiced by the locals. Thereafter, the roasted seeds were pulverized to fine particle using the laboratory mill (SPI Supplies, PA, USA) and then stored in an airtight container at 4–6°C until it was used.

### 2.4. Proximate Analysis

The moisture contents of the processed and unprocessed AYB seeds were determined after drying at 105°C according to the method of InduharaSwamy et al. [11]. The micro-Kjeldahl method was employed to determine the total nitrogen and the crude protein (N × 5.95) [12]. Crude lipids were extracted with petroleum ether, using a Soxhlet apparatus and ash contents (gravimetric) were determined based on methods outlined in AOAC [12]. Total carbohydrate was calculated by the difference method (summing the values of moisture, crude protein, ash, and crude fat (ether extract) and subtracting the sum from 100) [13]. Gross energy was calculated based on the formula by Eknayake et al. [14]:

(1) Gross  energy  (kJ  per  100 g  dry  matter)  =(crude  protein×16.7)   +(crude  lipid×37.7     +(crude  carbohydrates×16.7)).

Nitrogen free extract (NFE) was calculated by difference as NFE = total carbohydrate − crude fiber.

### 2.5. Mineral Analysis

Potassium, sodium, calcium, magnesium, manganese, phosphorus, copper, zinc, and iron in the samples of African Yam Bean seed were determined by X-ray spectrometric method (XRS). The mini pal 4 version PW 4030 X-ray Spectrometer (Perkin Elmer, Inc., USA) was used to determine the concentration of the elements in the samples. The mini pal 4 version PW 4030 X-ray Spectrometer is an energy dispersive microprocessor controlled analytical instrument designed for the detection and measurement of elements in a sample (solids, powders, and liquids), from sodium to uranium [15]. The samples were dried at 110°C until constant weights were obtained. The dried samples were weighed and manually ground using agate mortar and pestle (SPI Supplies, PA, USA). A binder (PVC dissolved in Toluene) was mixed with the sample to ensure that the sample is sufficiently thick enough to absorb the entire primary beam. This is followed by sieving using a turbulent mixer model which was aimed at homogenizing the samples in order to obtain a flat and representative sample surface and reduce the effect of surface irregularities. A flat and representative sample surface helps in maintaining a repeatable X-ray flux and ensuring that secondary X-rays from lighter elements often only emitted from the top few micrometres of the sample are captured. Pressure of about 7182 mmHg was applied to each sample mass of about 1.5 g to produce pellets of the samples for analysis. The pellets were loaded into the sample chamber of the spectrometer system.

The spectrometer was operated at a maximum voltage of 30 kV and a maximum current of 1 mA applied to produce the primary X-rays to excite the pellet. A preset time of 10 minutes (for each of the samples) was employed and an inbuilt Si (Li) detector was used for counting the secondary X-rays from the samples and the spectra from the samples were analysed by a PC running the dedicated Mini pal analytical software.

### 2.6. Antinutrient Analysis

Alkaline Titration Method [16] was used for hydrogen cyanide analysis while Reddy et al. [17] method was used for phytate, and the trypsin inhibitor analysis was done using spectrophotometric method as described by Arntfield et al. [18]. For tannin analysis, the method of Doss et al. [19] was used. The optical density (absorbance) readings were taken at 500 nm wavelength. Oxalate determination was carried out based on the method of Leyva et al. [20]. Solutions were prepared and read on the spectrophotometer at 440 nm.

### 2.7. Statistical Analysis

The analysis was carried out in triplicates for all determinations and the results of the triplicate were expressed as mean ± SEM. The SPSS 17.0 for Windows Computer Software Package was used for the Analysis of Variance (ANOVA) and the Pearson correlation coefficients. Significance of the differences was ascribed at *P* < 0.05 for ANOVA and *P* < 0.05 and 0.01 for Pearson correlation. The difference in means was compared using the Duncan's new Multiple Range test.

## 3. Results

### 3.1. Proximate Composition

Crude protein content for unprocessed AYB was significantly higher (*P* < 0.05) than the processed AYB (Table 1). Also, the moisture content showed significant (*P* < 0.05) differences among all the samples with the boiled sample having significantly higher values than the unprocessed sample and roasted sample. The levels of crude fibre in both processed samples were significantly (*P* < 0.05) lower than those of the unprocessed samples. The percentage of lipid composition was low in all the samples; however, the level of lipid in boiled sample was significantly lower (1.79 ± 0.03) than the unprocessed (2.84 ± 0.16) and the roasted samples (2.41 ± 0.06). The level of carbohydrate in roasted AYB was significantly (*P* < 0.05) higher than that in the unprocessed AYB. However, there was no significant difference (*P* > 0.05) between the levels of carbohydrate in the unprocessed sample compared to the boiled sample.

### 3.2. Antinutrients

The antinutrient content (oxalate, tannins, phytate, trypsin inhibitor, and hydrogen cyanide) was significantly (*P* < 0.05) higher in unprocessed sample compared to the processed sample (Table 2). In this work, roasting was found to have greater efficiency in the elimination or reduction of the levels of phytate available in the AYB while boiling seemed to eliminate oxalate more efficiently when compared with roasting.

### 3.3. Minerals

Roasting increased the percentage levels of Ca, K, Cu, Fe, Mn, Mg, P, and Na as follows: 0.41, 31.20, 11.48, 0.53, 66.87, 1.13, 44.39, and 10.51, respectively, while the percentage level of Zn was decreased by 38.35. Boiling, on the other hand, caused decrease in the percentage levels of Ca, K, Fe, Mn, and Zn as follows: 9.63, 8.54, 0.76, 8.06, and 38.02, respectively, while there was an increase in the percentage levels of Cu (5.74), Mg (8.88), P (18.56), and Na (12.31). In terms of significance, boiling reduced significantly (*P* < 0.05) the levels of Ca, K, and Zn compared to the unprocessed sample while roasting improved significantly (*P* < 0.05) the levels of Ca, K, Cu, Mn, P, and Na (Table 3). However, there was no significant difference (*P* > 0.05) between the level of Fe and Mg in both processed and unprocessed samples.

### 3.4. Correlation Analyses

The correlation matrix showed some important relationships between the chemical components analysed.  Table 4 showed the correlation matrix for unprocessed AYB in which case there was a perfect positive relationship (*r* = 1.000*) significant at the 0.05 level (2-tailed) between the crude protein and total protein for unprocessed sample. A near perfect negative relationship (*r* = −0.998) significant at the 0.05 level (2-tailed) was exhibited between ash and oxalate content. Similar relationship was exhibited by nitrogen-free extract with tannins. However, a near perfect positive relationship (*r* = 0.999) with significance at the 0.05 level (2-tailed) was exhibited by nitrogen-free extract with phytate. A similar relationship was exhibited by crude lipid with trypsin inhibitor (*r* = 0.999*), crude lipid with hydrogen cyanide (0.997*), and moisture with crude fibre (*r* = 0.997*).

For boiled AYB sample, crude protein exhibited a near perfect positive relationship (*r* = 0.999*) significant at the 0.05 level (2-tailed) with true protein (Table 5). Nitrogen-free extract was seen to show a perfect positive relationship with carbohydrate (*r* = 1.000**) with significance at the 0.01 level (2-tailed). Lipid and oxalate were seen to have a similar relationship with carbohydrate and trypsin inhibitor, respectively. However, a perfect negative relationship (*r* = −1.000**) significant at the 0.01 level (2-tailed) was exhibited between nitrogen-free extract and lipid. Regarding tannins and hydrogen cyanide, a near perfect negative relationship (*r* = −0.998*) significant at the 0.05 level (2-tailed) was observed.

Roasted AYB crude protein exhibited near perfect positive relationship with gross energy and oxalate (*r* = 0.999*) at significance of 0.05 level (2-tailed) (Table 6). Similar relationships were exhibited between crude fibre and carbohydrate, gross energy and tannins, and true protein and oxalate. However, a perfect negative relationship (*r* = −1.000**) significant at the 0.01 level (2-tailed) was shown by ash with tannins, while moisture exhibits a perfect negative relationship (*r* = −1.000*) significant at the 0.05 level (2-tailed) with tannins. Ash, nitrogen-free extract, moisture, and phytate all exhibited near perfect negative relationships significant at the 0.05 level (2-tailed) with gross energy (*r* = −0.997*), crude fibre (*r* = −0.999*), and trypsin inhibitor (*r* = −0.999*), respectively.

## 4. Discussion

The level of both the crude protein and true protein for the processed samples is lower than that of the raw sample which is in agreement with the report of Adegunwa et al. [21]. This reduction could be attributed to denaturation/leaching of the protein in the boiled sample and denaturation in the roasted sample. Due to the low fat content when compared with other nuts such as almonds, hazelnuts, and walnuts, AYB has low caloric value, which makes it an interesting healthy food; however, it also limits its use for commercial oil production. This is because high fat diets lead to increased blood cholesterol levels and heart attack [22]. The carbohydrate content of AYB seed was very high compared to results obtained for winged bean, soybean, peanut, cowpea, and chickpea [23]. The processed samples recorded higher carbohydrate content, which support an earlier report by Agiang et al. [24] that suggested that processing causes the granules to break down, softens the cellulose, and makes the starch more available. The nitrogen-free extract (NFE) for the raw AYB seeds was found to be lower when compared to previous reports on certain underutilized food legumes such as* Cassia floribunda* and* Tamarindus indica* [25, 26]. The moisture contents for all the samples, processed and unprocessed, fall within the recommended range of 0–13% as reported by James [27]. The lowest moisture content registered by roasted sample indicates that the seeds are better preserved by roasting compared to other treatments [28]. The crude fibre, which represents the amount of indigestible sugar present in the sample, is generally better than that from most seeds as well as* Sphenostylis stenocarpa* obtained in eastern Nigeria [21, 29]. Reports have shown that diets that are low in fibre are undesirable as they could cause constipation and such diets have been associated with diseases of colon like piles, appendicitis, and cancer [30].

Antinutritional factors are generally toxic and may negatively affect the nutrient value of seeds by impairing protein digestibility and mineral availability. However, they are heat labile and hence may be inactivated by processing methods involving heat generation. The results for effect of heat treatment on antinutrients observed in the current study were similar to the report of Pugalenthi et al. [26] that showed that boiling and roasting reduced trypsin inhibitor activity. This may be as a result of the enhanced leaching of these antinutrients in the boiled sample into the heated water and the denaturation of the protein, trypsin inhibitor, by high temperature due to roasting. Nwosu [31] indicated a significant reduction in phytate, tannins, and trypsin inhibitor contents following cooking which is in agreement with this research. In the same manner, tannin content of the AYB seeds and other legumes such as pigeon pea (*Cajanus cajan*) and cowpea (*Vigna unguiculata*) decreased with processing [32]. Phytic acid is considered an antinutrient because it forms insoluble complexes with minerals. The oxalate values for processed and unprocessed AYB were significantly lower than those previously reported for lima bean, pigeon pea, and jack bean [33]. The hydrogen cyanide (HCN) content of the unprocessed AYB was higher than that reported by Chikwendu [34] for groundbean. Generally, processing reduced the level of all the antinutrients analyzed to their permissible levels.

The amount of magnesium, potassium, phosphorus, sodium, calcium, zinc, copper, and manganese content of AYB differed appreciably from those for Bambara groundnut [8]. The results also indicate that sodium, potassium, iron, manganese, and copper were higher in the AYB sample when compared with those reported for soybean [35]. The values for potassium, phosphorus, magnesium, and calcium are higher in AYB when compared with other legumes such as soybean, winged bean, peanut and cowpea [36], which suggested that AYB could be a better source and/or alternative of these minerals. The levels of calcium, copper, iron, manganese, zinc, magnesium, and sodium in the processed AYB met the Recommended Dietary Allowance (RDA) of these minerals in infants. Only copper, iron, and manganese met the RDA for infants, children, and adults. However, in conjunction with other foods, the RDA for these other minerals will be met. The levels of potassium and phosphorus in the processed AYB do not seem to meet the dietary requirement for infants, children, or adults.

In the correlation analyses, the effect of the antinutrients on the nutrient compositions was emphasized. The correlation analyses for raw sample suggest that oxalate, tannin, trypsin inhibitor (TI), and hydrogen cyanide (HCN) will negatively affect the mineral level (ash), carbohydrate, and nitrogen-free extract (NFE). This will make these nutrients unavailable; however, only oxalate has a significant effect on mineral level which is in agreement with the report of Adegunwa et al. [21] that suggests that oxalate forms complexes with minerals. Phytate negatively affected protein content, lipid, and fibre, though not significantly. Oxalate, trypsin inhibitors, and HCN still seemingly affected the mineral level, NFE, and moisture in both boiled and roasted samples. Tannins affected only ash and moisture in roasted sample compared to the boiled sample where it affects protein, lipid, fibre, and energy level of the AYB. Phytate, on the other hand, affected protein and lipid for both boiled and roasted AYB. Processing, though it reduced the level of the antinutrients to permissible limits, some quantity of the nutrients could still not be available due to the presence of the antinutrients. This may offer some explanations to why processing does not seem, in some cases, to increase the level of nutrients.

## 5. Conclusion

The results of the present study indicated that the AYB seeds have good nutritional profile with high level protein, carbohydrate, lipid, minerals, and other nutrients comparable with that of other common legume grains. Processing drastically reduced the level of antinutrients in the AYB with minimal effect on the nutritional quality.

Therefore, in view of the nutrient availability, low antinutritional content, and the level of mineral elements after processing, the consumption of* Sphenostylis stenocarpa*, especially roasted seeds, could help combat the effect of malnutrition experienced in the northern Nigeria and developing countries across the world. Further works should be carried out on management of resources in terms of fuel consumption during boiling. We recommend also that research be further carried out to ascertain the sensory quality and study how much is being grown in the northern Nigeria and the economic implications of consuming the seed.

## Figures and Tables

**Table 1:** Proximate composition of processed and unprocessed African Yam Bean (*Sphenostylis stenocarpa*) sample based on dry mass of the seeds.

	Crude protein (%)	Ash (%)	Nitrogen-free extract (%)	Lipids (%)	Moisture (%)	Crude fiber (%)	CHO (%)	Gross Energy (KJ/100 g)	NPN (%)	True protein (%)
Raw	21.78 ± 0.17^b^	2.64 ± 0.06^a^	54.22 ± 0.55^a^	2.84 ± 0.16^c^	9.23 ± 0.20^b^	9.29 ± 0.27^b^	63.51 ± 0.35^a^	1530.36 ± 3.95^b^	1.95 ± 0.02^b^	19.83 ± 0.16^b^
Boiled	19.74 ± 0.42^a^	2.61 ± 0.69^a^	55.92 ± 0.44^b^	1.79 ± 0.03^a^	11.95 ± 0.07^c^	7.99 ± 0.02^a^	63.91 ± 0.42^a^	1464.51 ± 2.37^a^	1.56 ± 0.03^a^	18.18 ± 0.44^a^
Roasted	20.47 ± 0.35^a^	2.61 ± 0.07^a^	61.24 ± 0.13^c^	2.41 ± 0.06^b^	4.88 ± 0.44^a^	8.38 ± 0.26^a^	69.62 ± 0.13^b^	1595.49 ± 9.09^c^	2.09 ± 0.42^c^	18.38 ± 0.33^a^

Values are expressed as mean ± SEM. Different superscripts down the column are significantly different from each other at *P* < 0.05.

**Table 2:** Antinutrients composition of processed and unprocessed African Yam Bean (*Sphenostylis stenocarpa*) based on dry mass of the seeds.

	Oxalate (mg/kg)	Tannins (mg/g)	Phytate (mg/100 g)	Trypsin inhibitor (mg/100 g)	HCN (mg/kg)
Raw	8.07 ± 0.07^c^	18.09 ± 0.53^b^	429.30 ± 1.80^c^	6.67 ± 0.33^b^	224.03 ± 2.54^b^
Boiled	2.22 ± 0.13^a^	3.65 ± 0.32^a^	125.30 ± 0.42^b^	1.10 ± 0.06^a^	43.24 ± 0.98^a^
Roasted	3.17 ± 0.22^b^	3.45 ± 0.27^a^	121.16 ± 0.42^a^	1.64 ± 0.31^a^	48.08 ± 0.64^a^
Permissible limit	3–5 mg/kg	20 mg/g	250–500 mg	0.7–3.0 mg/100 g	50 mg/kg

Values are expressed as mean ± SEM. Different superscripts down the column are significantly different from each other at *P* < 0.05.

**Table 3:** Mineral composition of processed and unprocessed African Yam ean (*Sphenostylis stenocarpa*) in mg/100 g dry mass of the ground seeds.

Mineral	Boiled	Roasted	Raw	% change due to boiling	% change due to roasting	^ #^Recommended Dietary Allowance (RDA) in mg/day
Ca	220.95 ± 1.95^a^	245.5 ± 5.50^b^	244.5 ± 5.50^b^	↓9.63	↑0.41	210–800*, 800**, 1200***
K	152 ± 2.00^a^	218.06 ± 1.94^c^	166.2 ± 2.85^b^	↓8.54	↑31.20	400–3000*, 4700^∗∗, ∗∗∗^
Cu	2.58 ± 0.01^ab^	2.72 ± 0.02^b^	2.44 ± 0.08^a^	↑5.74	↑11.48	0.2–0.44*, 0.7–0.89**, 0.9***
Fe	13.1 ± 0.10^a^	13.27 ± 0.07^a^	13.2 ± 0.26^a^	↓0.76	↑0.53	0.27–11*, 8–15**, 10***
Mn	3.08 ± 0.13^a^	5.59 ± 0.71^b^	3.35 ± 0.25^a^	↓8.06	↑66.87	0.003–1.5*, 1.6–2.2**, 1.8–2.3***
Zn	3.75 ± 0.25^a^	3.73 ± 0.16^a^	6.05 ± 0.65^b^	↓38.02	↓38.35	2–5*, 11**, 15***
Mg	44.16 ± 0.84^a^	41.02 ± 0.02^a^	40.56 ± 1.44^a^	↑8.88	↑1.13	30–130*, 240–360**, 320–340***
P	33.28 ± 0.28^b^	40.53 ± 1.47^c^	28.07 ± 0.94^a^	↑18.56	↑44.39	100–500*, 1250**, 700***
Na	396 ± 2.00^b^	389.67 ± 0.67^b^	352.61 ± 3.40^a^	↑12.31	↑10.51	120*, 1500^∗∗, ∗∗∗^

Values are expressed as mean ± SEM. Different superscripts across the row are significantly different from each other at *P* < 0.05.

↑: signifying increase; ↓: signifying decrease.

^#^Culled from the United States Department of Agriculture (USDA) and may be assessed via http://www.nap.edu/.

*Infants; **children; ***adults.

**Table 4:** Correlations for unprocessed African Yam Bean (*Sphenostylis stenocarpa*) sample.

	CP	Ash	NFE	Lip	Mst	CF	CHO	GE	NPN	TP	Ox	Tan	Phy	TI	HCN
CP	1														
Ash	−0.400	1													
NFE	−0.603	0.972	1												
Lip	0.981	−0.571	−0.747	1											
Mst	0.025	−0.926	−0.813	0.219	1										
CF	0.098	−0.951	−0.853	0.290	0.997*	1									
CHO	−0.869	0.801	0.918	−0.949	−0.516	−0.577	1								
GE	0.894	0.053	−0.181	0.790	−0.425	−0.358	−0.556	1							
NPN	0.963	−0.139	−0.366	0.893	−0.244	−0.173	−0.705	0.981	1						
TP	1.000*	−0.427	−0.626	0.986	0.055	0.127	−0.884	0.880	0.955	1					
Ox	0.462	−0.998*	−0.986	0.626	0.898	0.928	−0.840	0.015	0.207	0.488	1				
Tan	0.662	−0.952	−0.997*	0.795	0.766	0.811	−0.946	0.255	0.436	0.684	0.971	1			
Phy	−0.566	0.982	0.999*	−0.716	−0.838	−0.876	0.900	−0.137	−0.324	−0.590	−0.993	−0.993	1		
TI	0.973	−0.602	−0.771	0.999*	0.257	0.326	−0.960	0.765	0.875	0.979	0.655	0.818	0.742	1	
HCN	0.992	−0.510	−0.697	0.997*	0.149	0.220	−0.924	0.832	0.923	0.996	0.568	0.749	−0.664	0.994	1

*Correlation is significant at the 0.05 level (2-tailed).

**Correlation is significant at the 0.01 level (2-tailed);

CP: crude protein; NFE: nitrogen-free extracts; Lip: lipids; Mst: moisture; CF: crude fiber; CHO: carbohydrate; GE: gross energy; NPN: nonprotein nitrogen; TP: true protein; Ox: oxalate; Tan: tannins; Phy: phytate; TI: trypsin inhibitor; HCN: hydrogen cyanide.

**Table 5:** Correlations for boiled African Yam Bean (*Sphenostylis stenocarpa*) sample.

	CP	Ash	NFE	Lip	Mst	CF	CHO	GE	NPN	TP	Ox	Tan	Phy	TI	HCN
CP	1														
Ash	−0.725	1													
NFE	−0.949	0.470	1												
Lip	0.948	−0.469	−1.000**	1											
Mst	−0.046	0.721	−0.272	0.273	1										
CF	0.916	−0.387	−0.996	0.996	0.359	1									
CHO	−0.950	0.474	1.000**	−1.000**	−0.268	−0.995	1								
GE	0.642	−0.994	−0.367	0.366	−0.795	0.280	−0.371	1							
NPN	−0.734	0.064	0.911	−0.911	−0.645	−0.945	0.909	0.049	1						
TP	0.999*	−0.696	−0.961	0.961	−0.005	0.931	−0.962	0.611	−0.761	1					
Ox	0.427	−0.932	−0.119	0.118	−0.923	0.027	−0.123	0.967	0.301	0.390	1				
Tan	−0.851	0.979	0.642	−0.641	0.563	−0.569	0.645	−0.949	0.268	−0.829	−0.838	1			
Phy	−0.535	0.970	0.240	−0.240	0.869	0.151	0.245	−0.991	−0.181	−0.500	−0.992	0.899	1		
TI	0.449	−0.941	−0.144	0.143	−0.913	0.052	−0.148	0.973	0.277	0.413	1.000*	−0.851	−0.995	1	
HCN	0.821	−0.988	−0.599	0.598	−0.608	0.523	−0.602	0.965	−0.215	0.797	0.866	−0.998*	−0.921	0.879	1

*Correlation is significant at the 0.05 level (2-tailed).

**Correlation is significant at the 0.01 level (2-tailed);

CP: crude protein; NFE: nitrogen-free extracts; Lip: lipids; Mst: moisture; CF: crude fiber; CHO: carbohydrate; GE: gross energy; NPN: nonprotein nitrogen; TP: true protein; Ox: oxalate; Tan: tannins; Phy: phytate; TI: trypsin inhibitor; HCN: hydrogen cyanide.

**Table 6:** Correlations for roasted African Yam Bean (*Sphenostylis stenocarpa*) sample.

	CP	Ash	NFE	Lip	Mst	CF	CHO	GE	NPN	TP	Ox	Tan	Phy	TI	HCN
CP	1														
Ash	−0.993	1													
NFE	−0.896	0.941	1												
Lip	0.941	−0.896	−0.693	1											
Mst	−0.996	1.000*	0.933	−0.906	1										
CF	0.875	−0.925	−0.999*	0.659	−0.915	1									
CHO	0.851	−0.906	−0.996	0.623	−0.896	0.999*	1								
GE	0.999*	−0.997*	−0.914	0.926	−0.999*	0.894	0.872	1							
NPN	0.539	−0.632	−0.857	0.222	−0.613	0.880	0.901	0.573	1						
TP	0.994	−0.975	−0.843	0.972	−0.980	0.817	0.790	0.989	0.445	1					
Ox	0.999*	−0.987	−0.875	0.955	−0.991	0.852	0.827	0.996	0.500	0.998*	1				
Tan	0.994	−1.000**	0.939	0.899	−1.000*	0.922	0.903	0.998*	0.626	0.977	0.988	1			
Phy	−0.637	0.544	0.228	−0.860	0.564	−0.183	−0.137	−0.604	0.307	−0.716	−0.671	−0.550	1		
TI	0.604	−0.508	−0.187	0.838	−0.528	0.141	0.096	0.570	−0.347	0.686	0.639	0.514	−0.999*	1	
HCN	0.972	−0.939	−0.768	0.994	−0.947	0.737	0.705	0.962	0.327	0.992	0.982	0.941	−0.799	0.773	1

*Correlation is significant at the 0.05 level (2-tailed).

**Correlation is significant at the 0.01 level (2-tailed);

CP: crude protein; NFE: nitrogen-free extracts; Lip: lipids; Mst: moisture; CF: crude fiber; CHO: carbohydrate; GE: gross energy; NPN: nonprotein nitrogen; TP: true protein; Ox: oxalate; Tan: tannins; Phy: phytate; TI: trypsin inhibitor; HCN: hydrogen cyanide.
